# HIV-1 Tat Triggers TGF-β Production and NK Cell Apoptosis that is Prevented by Pertussis Toxin B

**DOI:** 10.1080/17402520600645712

**Published:** 2006

**Authors:** Alessandro Poggi, Maria Raffaella Zocchi

**Affiliations:** 1 Laboratory of Experimental Oncology D National Institute for Cancer Research Genoa Italy; 2 Laboratory of Tumor Immunology Scientific Institute San Raffaele Milan Italy

## Abstract

Herein, we show that PTX-B and its non-toxic mutant PT9K/129G inhibit transcription and secretion of TGF-β elicited by HIV-1 Tat in NK cells. Moreover, Tat strongly activates the cJun component of the multimolecular complex AP-1, while TGF-β triggers cFos and cJun. Treatment of NK cells In turn,with PTX-B or PT9K/129G inhibits Tat and TGF-β-induced activation of AP-1. TGF-β enhances starvation-induced NK cell apoptosis, reduces the transcription of the antiapoptotic protein Bcl-2 and inhibits Akt phosphorylation induced by oligomerization of the triggering NK cell receptor NKG2D. All these TGF-β-mediated effects are prevented by PTX-B or PT9K/129G, through a PI-3K-dependent mechanism. Finally, PTX-B and PT9K/129G upregulate Bcl-x_L_, the isoform of Bcl-x that protects cells from starvation-induced apoptosis. Of note, in NK cells from patients with HIV-1 infection, mRNA expression of Bcl-2 and Bcl-x_L_ was consistently lower than that of healthy donors; interestingly, TGF-β and Tat were detected in the sera of these patients. These data suggest that Tat-induced TGF-β production and the consequent NK cell failure, possibly occurring during early HIV-1 infection, may be regulated by PTX-B and PT9K/129G.


*Clinical & Developmental Immunology*, June–December 2006; 13(2–4): 369–372

Taylor & Francis

Taylor & Francis Group

### HIV-1 Tat triggers TGF-β production and NK cell apoptosis that is prevented by pertussis toxin B

ALESSANDRO POGGI[1] & MARIA RAFFAELLA ZOCCHI[2]

[1]*Laboratory of Experimental Oncology D, National Institute for Cancer Research, Genoa, Italy, and* [2]*Laboratory of Tumor Immunology, Scientific Institute San Raffaele, Milan, Italy*

**Abstract**

Herein, we show that PTX-B and its non-toxic mutant PT9K/129G inhibit transcription and secretion of TGF-β elicited by HIV-1 Tat in NK cells. Moreover, Tat strongly activates the cJun component of the multimolecular complex AP-1, while TGF-β triggers cFos and cJun. Treatment of NK cells In turn,with PTX-B or PT9K/129G inhibits Tat and TGF-β-induced activation of AP-1. TGF-β enhances starvation-induced NK cell apoptosis, reduces the transcription of the antiapoptotic protein Bcl-2 and inhibits Akt phosphorylation induced by oligomerization of the triggering NK cell receptor NKG2D. All these TGF-β-mediated effects are prevented by PTX-B or PT9K/129G, through a PI-3K-dependent mechanism. Finally, PTX-B and PT9K/129G upregulate Bcl-$x_L$, the isoform of Bcl-x that protects cells from starvation-induced apoptosis. Of note, in NK cells from patients with HIV-1 infection, mRNA expression of Bcl-2 and Bcl-$x_L$ was consistently lower than that of healthy donors; interestingly, TGF-β and Tat were detected in the sera of these patients. These data suggest that Tat-induced TGF-β production and the consequent NK cell failure, possibly occurring during early HIV-1 infection, may be regulated by PTX-B and PT9K/129G.

**Keywords:** *TGF-β*, PTX-B, AP-1, PI-3K, NK cells

### Introduction

HIV-1 infection has been shown to induce production of several cytokines which, in turn, modulate the levels of HIV-1 expression in infected cells: this double-edged mechanism is supposed to play an important role in the pathogenesis of AIDS (Poli and Fauci 1993). HIV-1 products, among which endogenous Tat, induce the transcription of cytokines with immunosuppressive effects, including transforming growth factor (TGF)-β: regulation of TGF-β transcription by Tat has been claimed to contribute to immunosuppression in AIDS (Reinhold et al. 1999). We and others have reported on the possible immunosuppressive effects of extracellular Tat once taken up by bystander cells (Rubartelli et al. 1998; Zocchi et al. 1998; Poggi et al. 2002). Thus, a possibility exists that also exogenous Tat can induce TGF-β transcription in immunocompetent cells.

The transactivating effect of HIV-1 Tat is mediated by the multimolecular complex AP-1; interestingly, the same pathway is activated by TGF-β, which acts mainly upregulating the Jun family of AP-1 transcription factors (Gibellini et al. 1997; Cohen et al. 1999). In addition, TGF-β has been reported to induce apoptosis of different cell types through AP-1-dependent activation of SHIP which, in turn, determines the dephosphorylation of Akt, a phosphoinositol-3 kinase (PI-3K)-dependent enzyme that induces the transcription of the antiapoptotic proteins Bcl-2 and Bcl-x (Yamamura et al. 2000; Valderrama-Carvajal et al. 2002). The dephosphorylated form of Akt is inactive leading to down-regulation of Bcl-2 and Bcl-x transcription (Chao et al. 1998; Pugazhenthi et al. 2000). Suppression of TGF-β-induced apoptosis can be reached by upregulating the PI-3K/Akt pathway and pAkt that downregulates AP-1

Correspondence: A. Poggi, Laboratory of Experimental Oncology D, Largo R. Benzi 10, 16132 Genoa, Italy. Tel:39 10 5737211. Fax:39 10 354282. E-mail: alessandro.poggi@istge.it

ISSN 1740-2522 print/ISSN 1740-2530 online © 2006 Taylor & Francis
DOI: 10.1080/17402520600645712

activation (Chen et al. 1998), thus providing cells with a mechanism controlling both Tat and TGF-β-mediated effects.

*HIV-1 Tat induces TGF-β production, inhibited by PTX-B through the block of AP-1*

We found that synthetic Tat induces very early transcription and secretion of TGF-β in NK cells; interestingly, both transcription and secretion of TGF-β are inhibited by a short exposure (10 min) of NK cells to the PTX-B oligomer or to the non-toxic mutant of PTX, PT9K/129G (both at 1 nM), which can be safely administered *in vivo* (Del Giudice et al. 1999). As reported (Gibellini et al. 1997), Tat induces the activation of AP-1, preferentially involving cJun in NK cells; of note, this activation is inhibited by either PTX-B or by PT9K/129G. TGF-β also induces AP-1 (Yamamura et al., 2000), thus possibly creating a positive loop on its synthesis and secretion; we found that exposure of NK cells to 10 ng/ml of TGF-β leads to a strong activation of cFos and, to a lesser extent, of cJun; again, pre-treatment of NK cells with either PTX-B or PT9K/129G (1 nM) abolished cFos and strongly inhibited cJun activation. The blocking effect of PTX-B or PT-9K/129G on Tat or TGF-β-induced cJun activation was abolished by LY294002, suggesting that both PTX-B and its mutant act mainly through PI-3K activation (Figure 1 shows a proposed model of these interactions).

*PTX-B and PT9K/129G maintain Akt phosphorylation and Bcl-$x_L$/Bcl-2 trancription and inhibit TGF-β-mediated NK cell apoptosis*

It is known that TGF-β is a mediator of apoptosis in different cell types (Yamamura et al. 2000): we found that it is able to accelerate starvation-induced apoptosis in NK cells. Indeed, about 8% of NK cells were apoptotic, evaluated as $PI^+$ cells that identifies apoptotic cells (DNA content $<2n$), after 48 h of in the absence of growth factors (IL2), being approximately 35% in the presence of 10 ng/ml of TGF-β, while <10% of apoptotic cells were detected after pretreatment with PTX-B or with PT9K/129G, before exposure to TGF-β. Of note, NK cell apoptosis was also detected after 48 h of NK cells cultured in the presence of supernatants from autologous NK cells treated for 24 h with Tat, containing 20 ng/ml of TGF-β: this apoptosis was blocked by adding neutralizing anti-TGF-β mAb (5 μg/ml), further supporting a Tat-induced TGF-β-mediated autocrine pathway. We investigated the molecular mechanism whereby PTX-B antagonizes TGF-β: this cytokine is known to induce dephosphorylation of Akt, thus impairing the protection from apoptosis (Valderrama-Carvajal et al. 2002). Interestingly, PTX is an activator of PI-3K, which in turn leads to phosphorylation and activation of Akt (Pugazhenthi et al. 2000). In keeping with this, we found that the percentage of pAkt vs total Akt increased from 5 to 25% at 8 min,

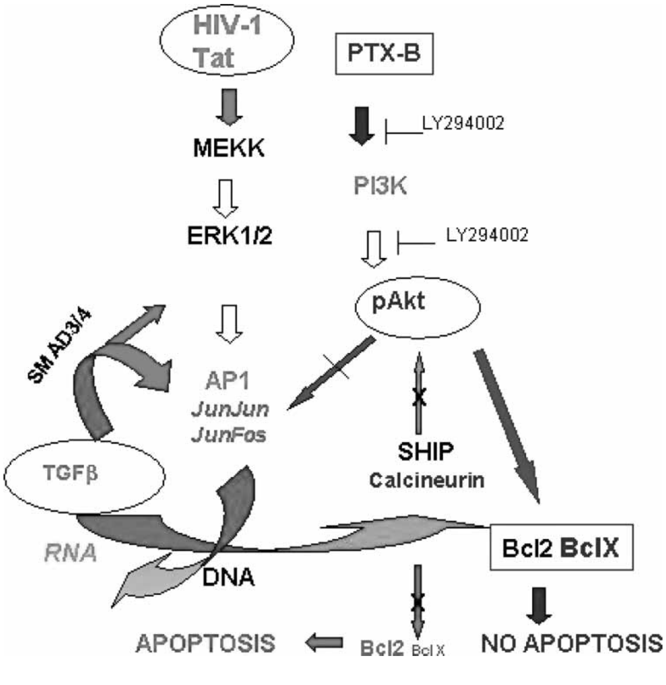

Figure 1. Proposed mechanism of action of Tat and TGF-β and role of PTX-B and PT-9K/129G. Tat induces ERK1/2 which activate AP-1, responsible for TGF-β production. TGF-β itself can activate ERK1/2 and AP-1 through SMAD3/4: both these steps are inhibited by ERK1/2 inhibitors. A further effect of TGF-β?is the dephosphorylation of Akt, elicited through a double mechanism: activation of SHIP and/or of calcineurin. The downregulation of Akt leads to a decrease in the synthesis of anti-apoptotic proteins such as Bcl-2 and Bcl-x. On the contrary, PTX-B or its non-toxic mutant PT9K/129G activates PI-3K maintaining the phosphorylated form of Akt and the synthesis of antiapoptotic proteins: this pathway is blocked by PI-3K inhibitors, such as LY294002.

upon treatment of NK cells with 1 nM PTX-B or PT9K/129G: this effect was blocked in the presence of the PI-3K blocker LY294002, supporting the hypothesis that PTX-B acts through the activation of PI-3K (Figure 1). Phosphorylated Akt is known to induce transcription of the antiapoptotic proteins Bcl-2 and Bcl-x; in particular, Bcl-$x_L$ is involved in the protection from starvation-induced apoptosis (Yamamura et al. 2000). TGF-β-induced apoptosis is the consequence of Akt dephosphorylation and this effect can be counteracted by upregulating the PI-3K-dependent Akt pathway (Yamamura et al. 2000; Valderrama-Carvajal et al. 2002). Accordingly, in NK cells exposed to TGF-β a reduction by 80% of Bcl-2 transcript was observed, while Bcl-$x_L$ mRNA was undetectable. Treatment with PTX-B or with PT9K/129G, not only prevented the inhibitory effect of TGF-β on Bcl-2, but also induced Bcl-$x_L$ transcription, even in the presence of TGF-β.

### *Bcl-2 and Bcl-$x_L$ transcription is down-regulated in NK cells from HIV-1 patients showing TGF-β and Tat in their Serum*

To verify the *in vivo* relevance of our findings, we studied 20 HIV-1 infected patients at stage A of the disease. We evaluated the mRNA for Bcl-2, Bcl-$x_L$ and Bcl-$x_S$ in purified NK cells and PBMC obtained from these patients, compared to NK cells and PBMC from healthy donors, matched for sex and age. As shown in table I, the level of Bcl-$x_L$ and Bcl-2 mRNA transcripts was consistently lower in purified NK cells (Table I) or in unfractionated PBMC (not shown) of HIV-1 infected patients than in healthy donors. TGF-β was present in the serum of all HIV-1 patients: in particular, in 13 out of 20 patients serum levels of TGF-β ranged between 50 and 100 ng/ml, while in healthy donors tested TGF-β was always $<10$ ng/ml ($n = 15$, Table I). In six patients, Tat was detectable in the serum at 10–50 nM concentration, and viremia was found (HIV-1 mRNA ranging between 1000 and 24,000 copies/ml) in eleven patients. Interestingly, regression analysis showed that RNA copy number is significantly associated with TGF-β ($r = 0.70$) and Tat ($r = 0.77$) serum levels. Moreover, in these patients the percentage of $CD3^-CD16^+$ (NK) cells was consistently lower (7 ± 3%) than in patients with low or undetectable TGF-β or Tat (14 ± 4%) or in healthy donors (15 ± 3%) (Table I).

### Conclusions

We have demonstrated that HIV-1 Tat induces both transcription and secretion of TGF-β which, in turn,

Table I. Increase of TGF-β and detection of HIV-1 Tat in the serum of patients with high HIV-1 and low Bcl-2/Bcl-$x_L$ mRNA

| Pt. | CD4/CD8 ratio* | $CD3^-CD16^+$ cells (%)* | Bcl-x $_L$/GAPDH %† | Bcl-2/GAPDH %† | HIV-1 RNA (copies/ml$^{-1}$)‡ | TGF-β (ng/ml)¶ | Tat (nM)§ |
|---|---|---|---|---|---|---|---|
| 1 | 0.2 | 4 | 1 (29) | 10 (30) | 24,000 | 95 | 40 |
| 2 | 0.5 | 5 | 2 (42) | 12 (22) | 17000 | 90 | 50 |
| 3 | 0.4 | 6 | 5 (36) | 9 (38) | 9000 | 100 | 50 |
| 4 | 1.0 | 4 | 4 (41) | 8 (44) | 8100 | 85 | 10 |
| 5 | 1.0 | 3 | 6 (40) | 11 (43) | 6800 | 80 | 20 |
| 6 | 0.5 | 7 | 2 (36) | 12 (26) | 6000 | 100 | 40 |
| 7 | 0.1 | 10 | 3 (45) | 10 (33) | 5000 | 105 | 30 |
| 8 | 0.8 | 8 | 2 (53) | 9 (23) | 2700 | 100 | 50 |
| 9 | 0.9 | 5 | 3 (51) | 8 (31) | 900 | 95 | 40 |
| 10 | 1.0 | 12 | 2 (48) | 9 (28) | 900 | 50 | 20 |
| 11 | 1.0 | 11 | 4 (36) | 10 (39) | 900 | 35 | 10 |
| 12 | 1.2 | 10 | 3 (40) | 8 (30) | 800 | 55 | 10 |
| 13 | 1.0 | 12 | 2 (50) | 12 (40) | <80 | 100 | 20 |
| 14 | 1.3 | 18 | 3 (38) | 11 (37) | <80 | 28 | n.d. |
| 15 | 1.7 | 14 | 2 (37) | 6 (29) | <80 | 20 | n.d. |
| 16 | 2.9 | 18 | 5 (37) | 8 (35) | <80 | 15 | n.d. |
| 17 | 1.2 | 12 | 4 (50) | 9 (40) | <80 | 80 | 10 |
| 18 | 1.1 | 15 | 5 (44) | 7 (34) | <80 | 20 | n.d |
| 19 | 1.5 | 13 | 4 (36) | 12 (46) | <80 | 15 | n.d. |
| 20 | 1.3 | 16 | 6 (42) | 10 (43) | <80 | 30 | n.d. |

* $CD4^+$ or $CD8^+$ and $CD3^-CD16^+$ cells were evaluated by immunofluorescence with the specific mAbs and FACS analysis. The percentage of $CD3^-CD16^+$ cells and CD4/CD8 ratio in the peripheral blood of 15 healthy donors tested for comparison was 15 ± 3 and 1.7 ± 0.4, respectively. † Densitometric analysis of Bcl-2 and Bcl-$x_L$ mRNA evaluated by PCR in purified NK cells from 20 HIV-1-infected patients or 10 healthy donors (in parenthesis) expressed as Bcl-2 or Bcl-$x_L$ percentage of GAPDH analysed in the same sample. ‡ HIV-1 RNA was quantitated using the commercial branched DNA (bDNA ultrasensitive Assay, Chiron) with a lower limit of detection of 50 RNA copies/ml. ¶ TGF-β has been measured in the sera of HIV-1 infected patients by an ELISA commercial kit. Results expressed as ng/ml referred to the standard. TGF-β in the sera of 15 healthy donors tested for comparison was 8 ± 2 ng/ml. § Tat was measured using a polyclonal rabbit anti-Tat antiserum (10 μg/ml) as capture antibody and a biotinilated rabbit anti-Tat antiserum (1 μg/ml) as detection antibody, followed by Av-HRP and by the specific substrate. Results expressed as nM referred to synthetic Tat (Tecnogen) used as standard. n.d.: not detectable. Tat measured in the sera of 15 healthy donors tested for comparison was <1 nM.

accelerates starvation-induced NK cell apoptosis, inhibits Akt phosphorylation and down-regulates Bcl-$x_L$ and Bcl-2 transcription. These mechanisms, if operating *in vivo*, would lead to either functional impairment or even elimination of cells involved in early anti-viral response. Of note, PTX-B and its non-toxic mutant PT9K/129G, are able to counteract all these biochemical events in NK cells, through a PI-3K/Akt-dependent mechanism (Figure 1). The pathological relevance of our data is supported by the finding that in NK cells from patients with early HIV-1 infection, Bcl-$x_L$ and, to a lesser extent, Bcl-2 mRNA expression was consistently lower than that of healthy donors (table I); moreover, TGF-β, and in two thirds of the cases Tat, was detected in the sera of these patients, at concentrations which are biologically active *in vitro* (table I). We cannot exclude that, in these patients, TGF-β is produced by other cells than NK cells, including antigen presenting cells, endothelial cells or stromal cells in the lymph nodes, where similar Tat-mediated effets might be operative as well, thus possibly amplifying the immunosuppressive effect. Nevertheless, administration of PT9K/129G, which is already approved for human use as a component of a vaccine against B. pertussis infection (Roberts et al. 1995; Del Giudice et al. 1999), as a component of a Tat-based vaccine in HIV-1-infected patients might be of interest, not only as an adjuvant but also as a component potentially able to interfere with HIV-1 replication (Alfano et al. 2000; 2001), with unwanted Tat and with cytokine-mediated immunosuppressive action [2–4], allowing the first anti-viral defense to be maintained.

### Acknowledgements

This work was partially supported by the Istituto Superiore di Sanità (National Program of Research on AIDS).

### References

Alfano M, Pushkarsky T, Poli G, Bukrinsky M. 2000. The B-oligomer of pertussis toxin inhibits human immunodeficiency virus type 1 replication at multiple stages. J Virol 74:8767–8770.

Alfano M, Vallanti G, Biswas P, Bovolenta C, Licenzi E, Mantelli B, Pushkarsky T, Rappuoli R, Lazzarin A, Bukrinsky M, Poli G. 2001. The binding subunit of pertussis toxin inhibits HIV replication in human macrophages and virus expression in chronically infected promonocytic U1 cells. J Immunol 166:1863–1870.

Chao DT, Korsmeyer SJ. 1998. Bcl-2 family: Regulators of cell death. Annu Rev Immunol 16:395–419.

Chen RH, Su YH, Chuang RL, Chang TY. 1998. Suppression of trasforming growth factor-beta-induced apoptosis through a phosphatidylinositol 3-kinase/Akt-dependent pathway. Oncogene 17:1959–1968.

Cohen SS, Li C, Ding L, Cao Y, Pardee AB, Shevac EM, Cohen DI. 1999. Pronounced acute immunosuppression *in vivo* mediated by HIV-1 Tat challenge. Proc Natl Acad Sci USA 96:10842–10847.

Del Giudice G, Rappuoli R. 1999. Genetically derived toxoids for use as vaccines and adjuvants. Vaccine 17(Suppl. 2):S44–S52.

Gibellini D, Caputo A, Capitani S, La Placa M, Zauli G. 1997. Upregulation of c-Fos in activated T lymphoid and monocytic cells by human immunodeficiency virus-1 Tat protein. Blood 89:1654–1664.

Poggi A, Carosio R, Spaggiari MG, Fortis C, Tambussi G, Dell'Antonio G, DalCin E, Rubarteli A, Zocchi MR. 2002. NK cell activation by dendritic cells is dependent on LFA-1-mediated induction of calcium-calmodulin kinase II: Inhibition by HIV-1 Tat C-terminal domain. J Immunol 168:95–101.

Poli G, Fauci AS. 1993. Cytokine modulation of HIV expression. Semin Immunol 5:165–173.

Pugazhenthi S, Nesterova A, Sable C, Heidenreich KA, Boxer LM, Reusch JE. 2000. Akt/Protein kinase B up-regulates Bcl-2 expression through cAMP-response element-binding protein. J Biol Chem 275:10761–10766.

Reinhold D, Wrenger S, Kahne T, Ansorge S. 1999. HIV-1 Tat: Immunosuppression via TGF-beta1 induction. Immunol Today 8:384–385.

Roberts M, Bacon A, Rappuoli R, Pizza M, Cropley I, Douce G, Dougan G, Marinaro M, McGhee J, Chatfield S. 1995. A mutant pertussis toxin molecule that lacks ADP-ribosyltransferase activity, PT-9K/129G, is an effective mucosal adjuvant for intranasally delivered proteins. Infect Immun 63:2100–2108.

Rubartelli A, Sitia R, Poggi A, Zocchi MR. 1998. HIV-1 Tat: A polypeptide for all seasons. Immunol Today 19:543–545.

Valderrama-Carvajal H, Cocolakis E, Lacerte A, Lee EH, Krystal G, Ali S, Lebrun JJ. 2002. Activin/TGF-beta induce apoptosis through Smad-dependent expression of the lipid phosphatase SHIP. Nat Cell Biol 4:963–969.

Yamamura Y, Hua X, Bergelson S, Lodish HF. 2000. Critical role of Smads and AP-1 complex in transforming growth factor-beta-dependent apoptosis. J Biol Chem 275:36295–36302.

Zocchi MR, Rubarteli A, Morgavi P, Poggi A. 1998. HIV-1 Tat inhibits human natural killer cell function by blocking L-type calcium channels. J Immunol 161:2938–2943.

